# Evaluation of Excitation Propagation in the Rabbit Heart: Optical Mapping and Transmural Microelectrode Recordings

**DOI:** 10.1371/journal.pone.0123050

**Published:** 2015-04-16

**Authors:** Regina Mačianskienė, Irma Martišienė, Antanas Navalinskas, Rūta Vosyliūtė, Rimantas Treinys, Birutė Vaidelytė, Rimantas Benetis, Jonas Jurevičius

**Affiliations:** Institute of Cardiology, Lithuanian University of Health Sciences, Kaunas, Lithuania; University at Buffalo, UNITED STATES

## Abstract

**Background:**

Because of the optical features of heart tissue, optical and electrical action potentials are only moderately associated, especially when near-infrared dyes are used in optical mapping (OM) studies.

**Objective:**

By simultaneously recording transmural electrical action potentials (APs) and optical action potentials (OAPs), we aimed to evaluate the contributions of both electrical and optical influences to the shape of the OAP upstroke.

**Methods and Results:**

A standard glass microelectrode and OM, using an near-infrared fluorescent dye (di-4-ANBDQBS), were used to simultaneously record transmural APs and OAPs in a Langendorff-perfused rabbit heart during atrial, endocardial, and epicardial pacing. The actual profile of the transmural AP upstroke across the LV wall, together with the OAP upstroke, allowed for calculations of the probing-depth constant (k ~2.1 mm, n = 24) of the fluorescence measurements. In addition, the transmural AP recordings aided the quantitative evaluation of the influences of depth-weighted and lateral-scattering components on the OAP upstroke. These components correspond to the components of the propagating electrical wave that are transmural and parallel to the epicardium. The calculated mean values for the depth-weighted and lateral-scattering components, whose sum comprises the OAP upstroke, were (in ms) 10.18 ± 0.62 and 0.0 ± 0.56 for atrial stimulation, 9.37 ± 1.12 and 3.01 ± 1.30 for endocardial stimulation, and 6.09 ± 0.79 and 8.16 ± 0.98 for epicardial stimulation; (n = 8 for each). For this dye, 90% of the collected fluorescence originated up to 4.83 ± 0.18 mm (n = 24) from the epicardium.

**Conclusions:**

The co-registration of OM and transmural microelectrode APs enabled the probing depth of fluorescence measurements to be calculated and the OAP upstroke to be divided into two components (depth-weighted and lateral-scattering), and it also allowed the relative strengths of their effects on the shape of the OAP upstroke to be evaluated.

## Introduction

Within the past decade, optical mapping (OM) using noninvasive recordings of transmembrane action potentials (APs) has almost replaced the classical electrophysiological techniques that have been used to measure the propagation of electrical excitation in the heart [1–5]. However, a limitation of this technique is signal distortion due to scattering of fluorescent photons in excited tissue [1]; the scattering blurs the optical signals [6]. This especially occurs when near-infrared (NIR) voltage-sensitive dyes are used [7] because under such circumstances, fluorescent signals originate from a deep three-dimensional volume (scattering volume) of tissue located beneath the detection site [8]. This results in prolongation of the optical action potential (OAP) upstroke as well as in an increase in the width of the optically recorded excitation wavefront [7,8]. The blurring and prolongation of the OAP upstroke, possibly, are not accidental because the optical signal is an image created from a propagating electrical signal. Certainly, the OAP upstroke contains detailed information about electrical wave in the heart; however, this information could not be extracted easily. To ascertain how such information is coded in the OAP upstroke, in our opinion, the optical and electrical signals should be recorded simultaneously and independently. This could facilitate a better understanding of the OAP signal, which is used to investigate complex electrical activity in the heart.

To date, in the majority of electrophysiological studies, electrical AP recordings have been obtained from single cells [9] or from the epicardial surface of the heart [10–12]. For endocardial recordings, dissected tissue/wedge preparations [10,13] have been used to record electrical signals using multi-electrode-array/suction/floating microelectrodes [3,13–15]. For transmural electrical activity recordings in whole-heart preparations, needles/optrodes have been used [2,3,10,13]. Undoubtedly, all of these techniques can be used to determine or evaluate the duration of APs or of the repolarization process, but none of them can provide ideal reproductions of the upstroke of transmural APs. However, standard glass microelectrodes, which induce minimal cell damage, are not commonly used alongside OM [11,16,17] in whole heart preparation. Currently, the OAP upstroke has mainly been investigated using OM together with computer modeling [1,5,8,18], with the AP upstroke obtained from the epicardium used as a comparison.

Here, we introduce a new approach for analyzing electrical activity in OM studies through the recording of transmural APs, and comparing them with the OAPs obtained using the near-infrared di-4-ANBDQBS dye. In particular, we used the actual profile of the transmural AP upstroke to separate the electrical and optical contributions (from dyes and tissues) to the shape of the OAP upstroke, and this procedure enabled us to split the OAP upstroke into two components, which we termed the depth-weighted (DWTAP) and lateral-scattering (LSCATT) components. DWTAP and LSCATT correspond to the parts of the electrical propagating wave that are transmural and parallel to the epicardium, respectively. In this study, to evaluate the influence of both DWTAP and LSCATT on the OAP upstroke shape, we also needed to determine the probing-depth constant of the fluorescence signal that was detected directly during the OM experiment.

## Materials and Methods

All procedures conformed to European Community guiding principles and were approved by the State Food and Veterinary Service of the Republic of Lithuania (Permit Number: 0227) and the Ethics Committee of the Lithuanian University of Health Sciences.

### Isolated Langendorff-perfused rabbit hearts

**Fig 1:**
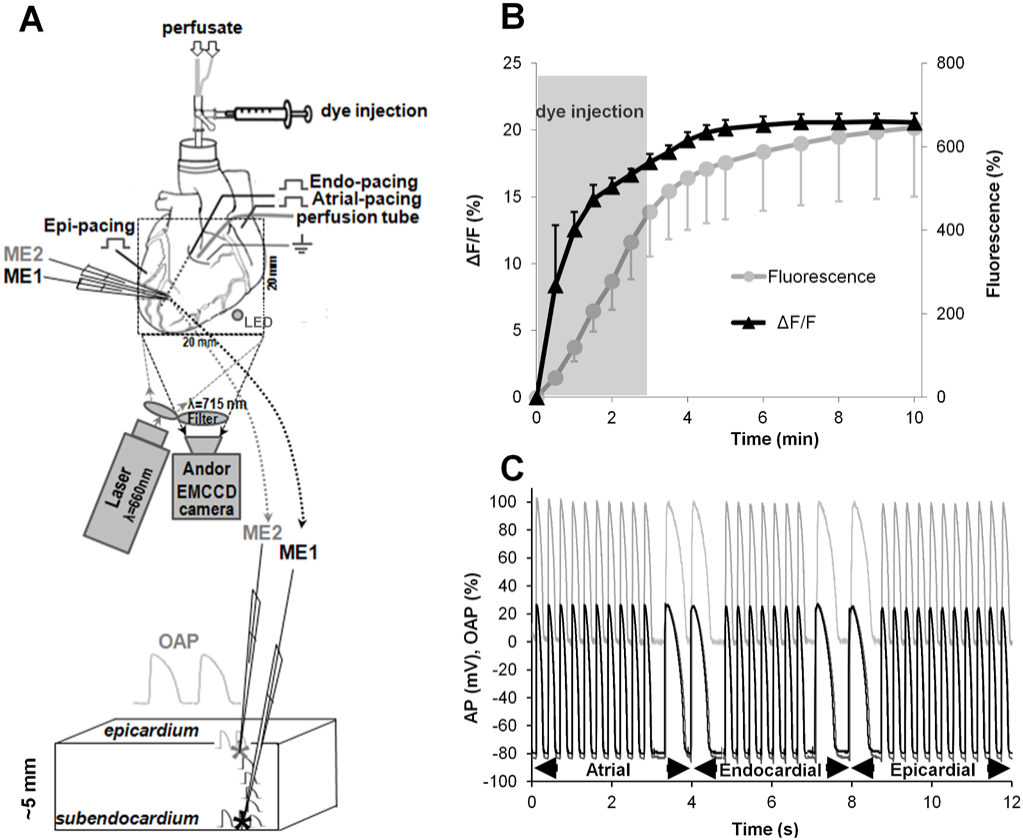
Design of the experimental system and data acquisition. (A) Instrumentation for synchronized recording of an OAP and an AP; ME1 and ME2 are the microelectrodes for the subendocardial and epicardial AP recordings. Their locations are indicated by asterisks. A square (dotted line) indicates the field of view of the camera. (B) Intensity changes in the voltage-sensitive signals (ΔF/F, black triangles), the fluorescence level at rest (gray circles) and the time at which dye was injected (gray area). (C) The stimulation protocol with automatic atrial-to-endocardial-to-epicardial pacing switchovers for OAP (normalized upper) and subendo-/epi-AP (in mV lower) recordings.

Experiments were performed on male New Zealand white rabbits (weight: 3.26 ± 0.27 kg, n = 14). To calm the animals prior to anesthetization, 10 mg/kg xylazine was injected subcutaneously. After 15–20 minutes, the animal was also anesthetized with an intravenous injection of ketamine (10 mg/kg) and heparin (1,000 U/kg) via the marginal ear vein. Then, thoracotomy was performed, and the heart was quickly excised and cannulated through the aorta. Using oxygenated Tyrode’s solution (see the composition below), coronary perfusion was restored within a few minutes using a Langendorff perfusion system with a constant pressure of ~80 mmHg. The coronary flow was 38.3 ± 1.6 mL/min (n = 14). Thereafter, several procedures were performed on the heart (Fig 1A). The following were inserted through a cut in the left atrium into the cavity of the left ventricular (LV) chamber: a bipolar silver electrode for stimulation of the endocardium, an AgCl reference electrode for microelectrode recordings, and a tube for additional perfusion with Tyrode’s solution to prevent the temperature from falling. The excess perfusate allowed the epicardial surface of an air-insulated heart to remain wet. Stimulation was performed from the following locations using the protocol shown in Fig 1C: 1) the right atrium, to evaluate wave propagation from the endocardial to the epicardial (endocardial-to-epicardial) surface (i.e., transmural propagation); 2) the endocardium, to evaluate the propagation both from the endocardial-to-epicardial surface and parallel to the epicardial surface; and 3) the epicardium, to evaluate propagation that is more parallel to the epicardial surface (i.e., lateral propagation). For atrial and epicardial stimulation, bipolar hook electrodes were embedded in the right atrium and the LV epicardial surface, respectively. The heart was continuously paced at 300 ms intervals with a 2 ms pulse width set at twice the diastolic threshold.

### Solutions and chemicals

Tyrode’s solution contained the following components (in mmol/L): 135 NaCl, 5.4 KCl, 1.8 CaCl_2_, 0.9 MgCl_2_, 0.33 NaH_2_PO_4_, 10 glucose, and 10 HEPES (pH 7.4 adjusted with NaOH, 37 ± 0.5°C). Both 20 μmol/L (±)-blebbistatin and 5 mmol/L 2,3-butanedione monoxime (BDM) were added to the perfusate to minimize motion artifacts. The heart was stained with a 10 mL slow bolus injection of the voltage-sensitive dye di-4-ANBDQBS (50 μmol/L) into the perfusate. To increase the staining efficiency, we reduced the perfusion rate to 50% of its normal value during dye loading (for ~3 minutes) by switching the superfusion solution to a tube with a smaller diameter. The overall fluorescence (Fig 1B) over the background (before staining) was calculated as a percentage. The efficiency of staining was evaluated by calculating the percentage change of the voltage-sensitive fraction of fluorescence (ΔF/F). ΔF/F increased along with the fluorescence and reached 20.38 ± 0.63% (n = 4), which indicated that a good-quality OAP signal had been achieved. The di-4-ANBDQBS dye (JPW-6033) was obtained from Dr. L. Loew (University of Connecticut, USA), (±)-blebbistatin was obtained from Cayman (USA), and all other chemicals were purchased from Sigma-Aldrich.

### Microelectrode recordings

Very thin and sufficiently long glass microelectrodes (MEs) filled with KCl (3 mol/L) were impaled in the LV wall from the epicardial surface (ME1 and ME2 in Fig 1A). The thickness of the LV wall where the MEs were impaled was 5.1 ± 0.2 mm (n = 14). In our experiments, the ME resistance ranged from 35 to 55 MΩ. One microelectrode (ME2) was used to impale an epicardial cell, while the other one (ME1) was moved in the transmural direction until it reached the subendocardium. The depth of impalement was controlled by hydraulic micromanipulators and special depth detectors (Millitron, Measuring Probes 1310) with an accuracy of ≤0.1 microns, and the depth detectors were directly connected to the glass microelectrode holders. The signal from these detectors was recorded in the LabChart8 Pro program simultaneously with electrical APs. The zero point for depth measurements was set when the microelectrodes reached the epicardial surface. APs were recorded using a MEZ-7101 two-channel amplifier (Nihon-Kohden, Japan) with capacitance compensation and high input impedance. Signals were digitized using a 16-channel PowerLab system (ADInstruments, Oxford, UK) at a frequency of 10-20 kHz.

### Optical recordings

The optical mapping setup was described previously [17]. Briefly, optical recordings were acquired using a cooled (-100°C) fast 14-bit EMCCD camera (iXon^EM+^DU-860, Andor Technology, Ireland) equipped with a 50 mm focal length objective. For the excitation of di-4-ANBDQBS, we used a 660 nm, 600 mW diode laser (SDL-660-600T, Shanghai Dream Lasers Technology) with a diffuser to homogeneously illuminate the heart. Fluorescence was collected through a 715 nm long-pass filter (NT46-066, Edmund Optics, USA) placed in front of the camera. To mark the time of electrical stimulation in the optical recording, a small LED (940 nm; Fig 1A) that generates 2 ms pulses in synchrony with the pacing cycle was placed in the field of view of the camera. The mapping field was 20 x 20 mm. Optical movies were acquired at a frame rate of 500 Hz with a resolution of 128 x 128 pixels (Fig 1A, square) using imaging software (Andor SOLIS x-3467) and an illumination intensity of ~0.5 mW/mm^2^. In parallel, as a control, we measured OAPs with faster frame rates (2000 Hz and 4000 Hz); these measurements were obtained in horizontal strips (at a resolution of 5-10 x 128 pixels) in the same area of the LV wall where the glass microelectrodes were inserted. The laser illumination time was controlled by a shutter, which opened and closed in synchrony with the camera. The movies were preprocessed using ImageJ 1.4S software. In routine experiments, OAPs were obtained from 5 x 5 pixels. The signal-to-noise ratio for optical action potentials under our experimental conditions (obtained from 5 x 5 pixels and 500 frames/s) was 40.58 ± 1.65 dB (n = 8). In some experiments, OAP signals were acquired from one pixel (~156 x 156 microns), i.e., almost from the length of a single cell, and at higher frequency rates to verify that the optical signals were not influenced by the measurement area or slow sampling rate (see S1A and S1B Fig).

### Light penetration measurements

In additional control experiments, the transmural light penetration along the LV wall was measured in cut-open Langendorff-perfused rabbit heart preparations using Tyrode’s solution supplemented with the same contraction-uncoupling drugs. A cut was performed nearly through the center of the LV in a manner that would not damage the main coronary arteries, which were important for maintaining good perfusion of the myocardium, as was the case during routine experiments. The transmural view of the section was angled perpendicularly to the EMCCD camera. The LV wall was illuminated from the epicardial side using LEDs at either the excitation (660 nm) or emission (735 nm) wavelength as light sources. We used a previously described technique to measure the light penetration [4], with the exception that our experiments were performed on coronary-perfused whole hearts and not slab preparations. Exponential decay functions were fit to the experimentally detected attenuation profiles away from the epicardial surface of the LV wall. The decay constants of these fits were the penetration depths (in mm) for the excitation (*δ*_*ex*_) and emission (*δ*_*f*_) wavelengths. The weighting function was calculated using the penetration depth constants and assuming that the amount of fluorescence emitted from a particular depth is the product of the fluence rate *Φ* of the excitation light and the escape function *G* for the emission light, which can be described using Eqs (1) and (2), respectively [19]:

$$\phi (z)=C_{1}\exp(-z/\delta_{\mathrm{ex}})-C_{2}\exp(-z/\delta_{ex})$$ (1)

$$G(z)=C_{3}\exp(-z/\delta_{f})$$ (2)

where *Φ*(*z*) is the amount of light, or the fluence rate, at depth z; *δ*_ex_ is the penetration depth for the excitation light; *G*(*z*) is the intensity of fluorescence that escapes from depth *z* to reach the tissue surface; *δ*_f_ is the penetration depth for the escaping fluorescence; and *C*_1_, *C*_2_, and *C*_3_ are free variable parameters, which can be determined by experimental measurement and data fitting.

As indicated by Eq (1), a second term accounts for light scatting out near the surface of the tissue [20]. Accordingly, the weighting function (the product of the fluence and escape functions) does not result in a single exponential process. Therefore, the weighting function was fitted with a mono-exponential function whose decay constant was assumed to be the probing-depth constant (*k*).

### Analysis and statistics

LabChart8 Pro software (Oxford, UK) was used to analyze the upstroke duration (i.e., the time interval between 10 and 90% depolarization) and activation time of APs (i.e., the time interval between the stimulus and dV/dt_max_). OAP maps of the activation time (evaluated at 50% of the depolarization level) were constructed using custom-written Scroll 1.16 software based on PV-Wave (Dr. S. Mironov, University of Michigan).

A parameter that measures optical signal blurring (τ_opt_) was calculated as the ratio of the upstroke durations of OAP to the electrical potentials recorded on the epicardial surface.

In our experiments, OAP and AP signals were recorded at different sampling rates (2 ms and 0.1-0.05 ms, respectively). To obtain similar time resolutions for the two signals, the OAP upstroke was fitted using a 5-parameter logistic function (see S1C and S1D Fig), and the OAP upstroke was then resampled to a resolution of 0.1-0.05 ms.

Data are presented as means ± SEMs. A paired *t*-test or the Wilcoxon test was used to calculate the statistical significance of the difference between the OAP and the AP and the difference between an epicardial AP (epi-AP) and subendocardial AP (subendo-AP) for each type of pacing. An analysis of variance (ANOVA) or the Kruskal-Wallis test was used to compare the parameters obtained from atrial/endo-/epi-pacing. A difference was considered statistically significant when P <0.05.

## Results

### OAP upstroke “abridgement” between subendocardial and epicardial APs

First, we attempted to visualize the variability of OAP and AP upstrokes when different stimulations were applied. For this purpose, we used two glass microelectrodes, which were inserted in the same area at the LV wall, for simultaneous recordings of electrical APs from the epicardium (epi-AP) and subendocardium (subendo-AP). This procedure allowed us to set "bounds" on part of the OAP upstroke and to reveal discrepancies between these signals.

**Fig 2:**
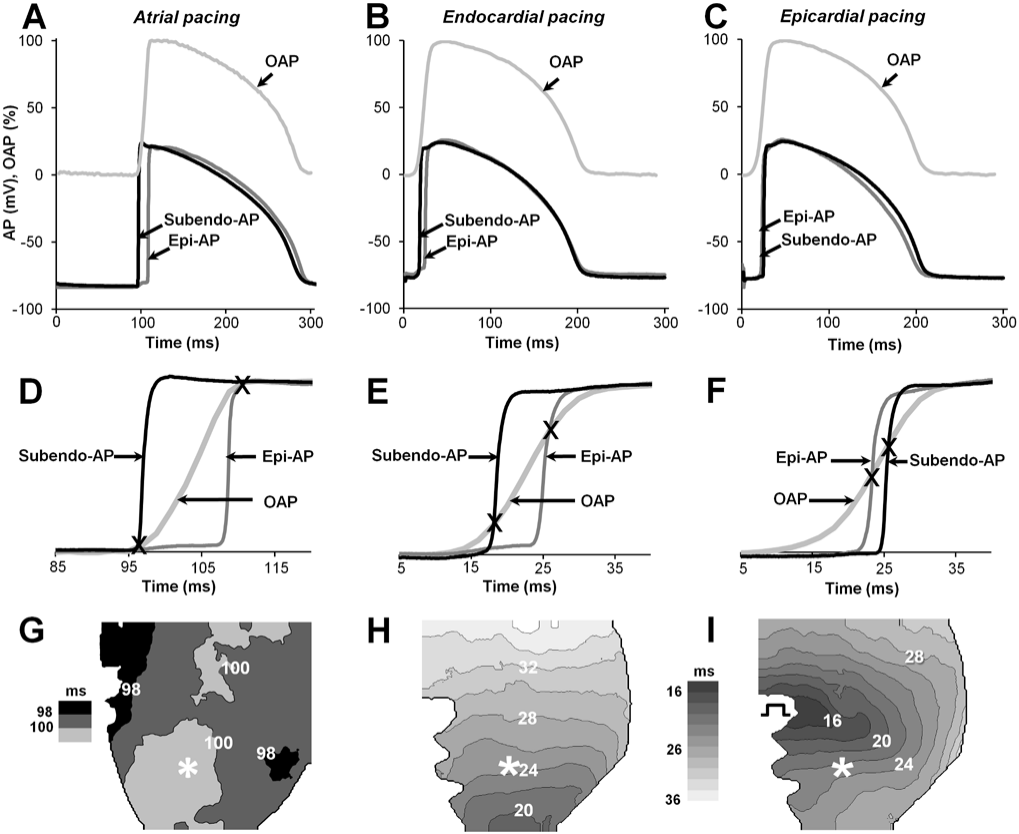
Characterization of optical and electrical APs using various stimulations of a whole rabbit heart. **(**A-C) Examples of simultaneous recordings of an OAP (light gray) and two APs, epicardial (gray) and subendocardial (black). The start time of the OAP was synchronized with that of the electrical APs. **(**D-F) Superimposition of the upstrokes of the subendo-/epi-AP and OAP from A-C on an expanded time scale. The cross indicates the crossing point between the OAP and the APs obtained from subendo- and epicardium. (G-I) OAP activation-time maps for atrial/endocardial/epicardial pacing. The interval between the isochrones (black lines) is 2 ms. Numbers near isochrones show the activation time in ms. The asterisks and the square pulse indicate the location of the microelectrodes and the stimulating electrode, respectively, on the epicardial surface. Stimulation period: 300 ms.

Fig 2A–2C shows OAP (upper) and electrical APs recordings (lower) and their superimposed upstrokes on an expanded time scale (Fig 2D–2F) for stimulations of the atrium (left), endocardium (middle), and epicardium (right). The upstroke duration of the subendo-AP was marginally shorter (in ms: 1.07 ± 0.25, 1.61 ± 0.45 and 1.09 ± 0.22) than that of the epi-AP (2.17 ± 0.27 ms, 2.64 ± 0.23 ms and 2.44 ± 0.28 ms; n = 8 for each; P <0.05) when stimulated from the atrium, endocardium and epicardium, respectively. However, the OAP upstroke duration under the same experimental conditions was much longer (10.15 ± 0.29 ms, 12.38 ± 0.51 ms, and 14.26 ± 0.58 ms, respectively; n = 8; P <0.05). The difference in AP upstroke duration occurred because the OAP, which was measured using the di-4-ANBDQBS dye, was the result of the summation of propagating electrical signals obtained from multiple cells in the LV wall [8,16], while the AP was obtained from a single cell. The mean values of standard AP parameters are presented in S1 Table.

As shown in Fig 2D–2F, the electrical epi-/subendo-APs, which were obtained from opposite LV surfaces, seemingly sets "bounds" on a certain part of the OAP upstroke (see between crosses). Those records along with the corresponding activation time maps of the OAP (Fig 2G–2I) helped to reveal the inhomogeneity of electrical signal propagation. According to the map, when the atrium was stimulated (Fig 2G), almost simultaneous activation (in ~2 ms) of the myocardium was obtained because the spread of excitation via Purkinje fibers occurs at the same time from different directions within the LV wall [21]. Under such conditions, the electrical wave propagates only from the endocardium-to-epicardium [13], i.e., only transmural propagation, which is favored during normal cardiac excitation, occurs. In such cases, the upstroke of the OAP was recorded accurately between the subendo-AP and the epi-AP (Fig 2D). Similarly, when hearts were stimulated from the endocardium (~apical location), a nearly homogeneous spread of activation with the excitation travelling upward was observed in the map (Fig 2H) because the Purkinje fibers were also excited first [21]. However, under such circumstances, the electrical wave propagates not only perpendicularly to the epicardial surface, but also parallel to it [13]. Possibly because of the appearance of the component of the propagating wave parallel to the epicardial surface, the obtained OAP upstroke already no longer coincided with the electrical APs (see the region outside the crosses in Fig 2E). During epicardial stimulation, the profile of the propagating wave depends on the distance from the stimulus initiation [18]. Therefore, it was possible to select a situation (under our conditions, ~7 mm from the pacing electrode) in which the propagation of the excitation wave was almost parallel to the epicardial surface. This results in even larger differences between the optical and electrical AP upstrokes (Fig 2F) than during endo-pacing. With epi-pacing, the activation time map indicates that the propagation has an elliptical shape (Fig 2I), suggesting that the fiber orientation and the inhomogeneity of the myocardium have significant influences.

We purposefully selected three completely different pacing conditions in which the direction of wave propagation varies; thus, we created conditions for excitation propagation similar to those that might appear in the myocardium.

### The transmural profile of the AP activation time

To evaluate the actual profiles of electrical wave propagation and its influence on the OAP upstroke, we obtained transmural recordings of electrical APs via multiple cell layers at the LV wall. For this purpose, one microelectrode was moved progressively from the epicardium to the endocardium in a direction that was almost perpendicular to the LV wall.

**Fig 3:**
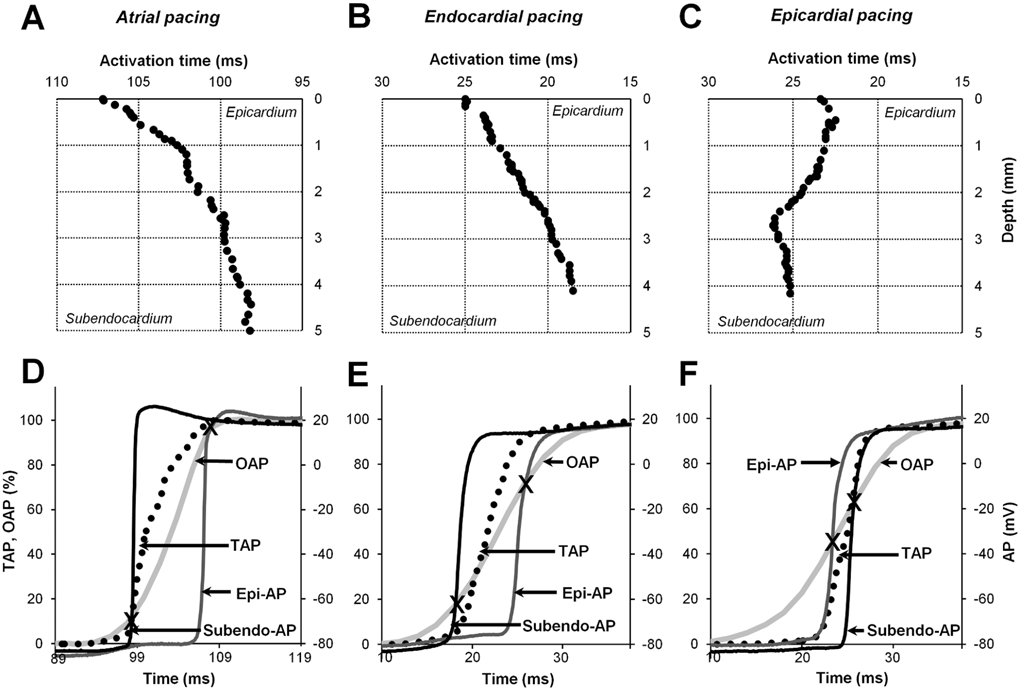
Transmural detection of electrical APs in the LV. (A-C) Transmural microelectrode recordings of the AP activation time at various depths during atrial/endocardial/epicardial pacing. (D-F) Superimposition of upstrokes: OAP (light gray line), subendo-AP (black line), epi-AP (gray line), and TAP (solid dotted line). The OAP and TAP were normalized. Note that the TAP does not coincide with the OAP. The data for this figure were obtained from the same experiments described in Fig 2.

Fig 3A–3C shows the change in the activation time of the APs as a function of depth in the LV wall for stimulations from the atrium, the endocardium, and the epicardium. In each experiment, 30 to 74 original AP recordings (48 on average) from different depths but separate cells in the LV wall, between two surfaces (epicardial/subendocardial), with spacings of 94.6 ± 11.3 μm, 116.5 ± 20.6 μm and 117.8 ± 19.7 μm for atrial, endocardial, and epicardial pacings, respectively, were obtained (n = 8 for each). From the shapes of these curves, we can suggest that the electrical wave propagation in the LV tissue is more or less non-uniform, possibly because of the influence of various morphological structures [10,22].

Thereafter, transmural AP (TAP) signals, which were calculated from the average data of the upstrokes of all normalized APs (see S2A–S2C Fig) across the LV wall (between the epicardium and the endocardium), were calculated for each pacing type (dotted trace in Fig 3D–3F). Obviously, the calculated TAP upstroke duration was much longer than the duration of the electrical APs recorded from the epi-/subendocardial surfaces of the myocardial wall. Nevertheless, the TAP upstroke still does not coincide with the OAP upstroke.

### OAP upstroke components and the probing-depth constant

It is known that because of the scattering phenomenon (dye/tissue), the fluorescence signal causes the OAP upstroke to be blurred [8]. Having calculated the transmural AP of the electrical wave propagation across the LV wall, we further managed to evaluate both the impact of the fluorescence light as it decays with increasing depth and the influence of lateral wave propagation on the OAP upstroke. Consequently, the calculation of TAPs enabled splitting of the OAP upstroke into two components, the depth-weighted TAP (DWTAP) and lateral-scattering (LSCATT), which correspond to the changes in the transmural electrical signal with depth and the component parallel to the epicardial surface, respectively. It should be noted that the method by which the probing-depth constant was detected, which was used to calculate the DWTAP component, is described below. Thus, we explored the interrelationship of these components, which constitute the OAP upstroke for each pacing type. Given the assumption that the sum of these components (DWTAP plus LSCATT) constitutes the OAP upstroke, LSCATT could be calculated by subtracting DWTAP from OAP.

**Fig 4:**
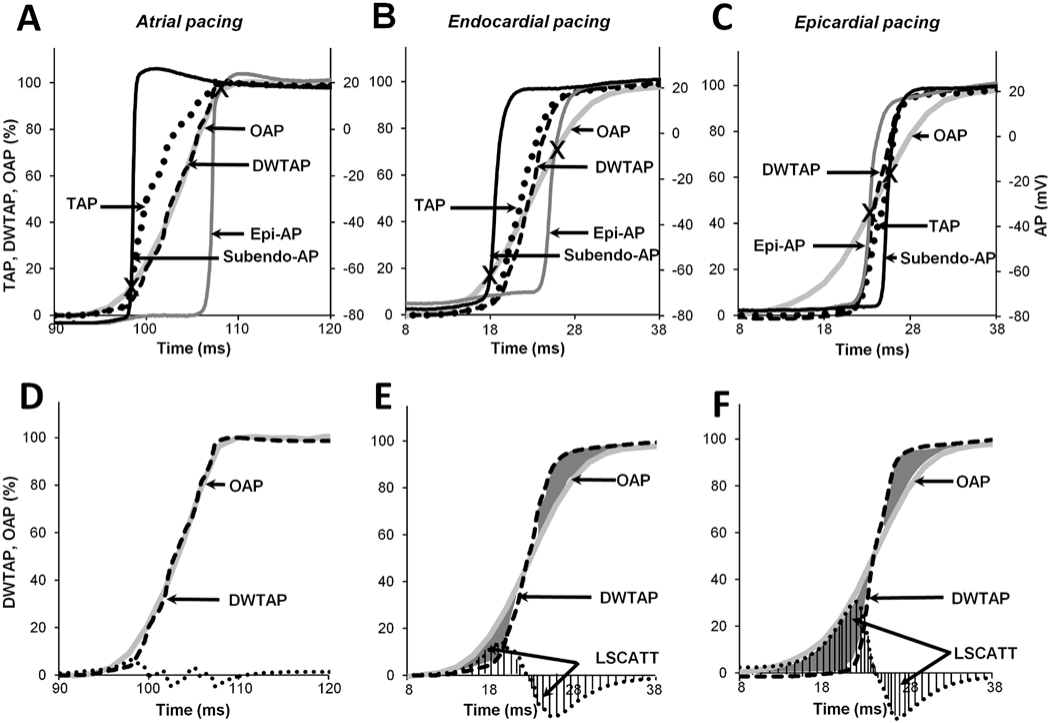
Influence of DWTAP and LSCATT on the OAP upstroke. (A-C) Traces of all upstrokes obtained for atrial/endo-/epi-pacing: simultaneous recordings of a subendo-AP (black line), epi-AP (gray line), and an OAP (light gray line) compared with the calculated TAP (dot line) and DWTAP (dashed line) signals. (D-F) Comparison of the OAP and DWTAP (same as in A-C). The difference between these signals provided the LSCATT (gray area); for better visualization, this LSCATT is also shown along the x-axis (dashed area).Note that a positive peak and a negative peak indicate an oncoming and receding wave, respectively, induced by LSCATT only when stimulated at the endo- or epicardium but not when stimulated at the atrium. DWTAP and LSCATT coexist around the mid-part of the OAP upstroke.

Fig 4A–4C shows the upstroke shapes in detail for APs obtained for three different types of pacing. When stimulated from the atrium, the DWTAP and OAP upstrokes highly coincided because under such experimental conditions, LSCATT is almost negligible (Fig 4D). However, as shown in Fig 4E and 4F, during endo-/epicardial stimulation, the calculated DWTAP upstrokes were still not equivalent to OAP. This finding indicates that the remaining part (Fig 4E and 4F, gray area or shaded area) of the OAP upstroke could be attributed to LSCATT. The largest difference between the OAP upstroke and DWTAP was obtained during the epi-pacing, apparently because a large lateral-scattering component occurred under such circumstances.

LSCATT was calculated using the integrated absolute values of the differences between the OAP upstroke and the DWTAP using a decay constant *k* for the probing depth, which reflects the capacity to measure the fluorescence according to the depth of the myocardium:

$$LSCATT(k)=\int_{tb}^{te}\left|OAP(t)-DWTAP(t,k)\right|dt$$ (3)

where *tb* and *te* are the time at the beginning and the end of the OAP upstroke, respectively. DWTAP was calculated as

$$DWTAP(t,k)=\int_{0}^{Z}AP(t,z)\exp(-z/k)dz$$ (4)

where *Z* is the maximal depth at which the AP was recorded (i.e., subendo-AP), and *z* = 0 denotes the epicardial surface.

These Eqs (3 and 4) show that both components depend on the magnitude of *k*. Accordingly, a change in the magnitude of *k* induces an alteration in the ratio between the DWTAP and LSCATT components.

**Fig 5:**
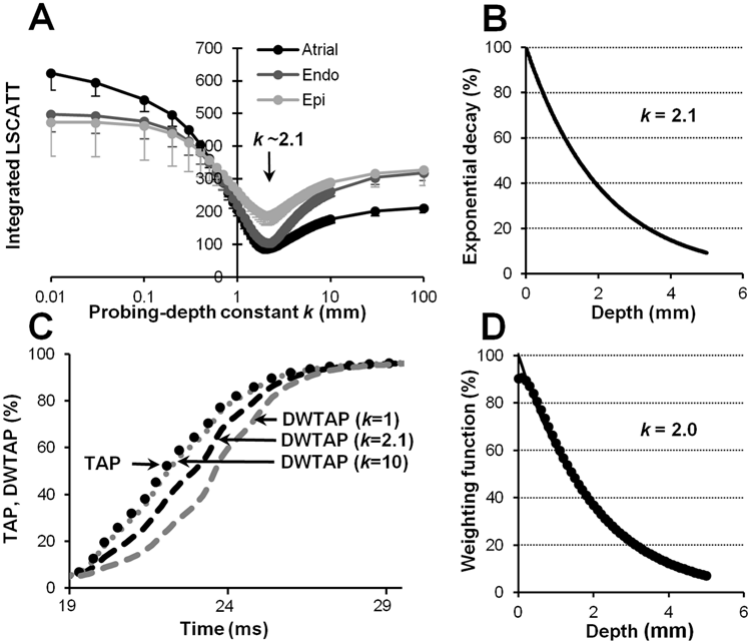
Detection of the probing-depth constant. (A) Dependence of LSCATT on the probing-depth constant of the fluorescence measurement for stimulations in the atrium (black line), endocardium (gray line), and epicardium (light gray line; n = 8 for each). The arrow indicates the minimum level of the integrated LSCATT when *k* = 2.1. The integrated LSCATT is given in units of the fluorescence intensity calculated as a percentage and multiplied by ms. (B) The exponential decay of the dependence of fluorescence probing on the depths under routine experiments was calculated using the formula *exp(-z/k)*, where *z* is the depth of the myocardium (mm), and *k* = 2.1. (C) Averaged transmural upstrokes: traces of a calculated TAP (dotted line) versus the DWTAP, when *k* = 1 (dashed gray line), 2.1 (dashed black line), and 10 (short-dotted line). (D) The data points (from separate measurements of penetration depths) for the weighting function (filled circles) for the red excitation light (660 mm) and the near-infrared emission (735 mm) light are shown, together with a curve (solid line) depicting single exponential decay with a constant *k* = 2 mm, which was obtained by fitting the data for the weighting function.

To evaluate the influence of *k* on the magnitude of LSCATT, we used increasing values of *k* (from 0.01 to 100 mm) in Eqs (3) and (4), and the interdependence was obtained for changes in the magnitude of LSCATT versus the probing-depth constant (Fig 5A).

As shown in Fig 5A, the curves from the obtained interdependence demonstrated a characteristic minimum independent of the type of pacing that was used. This minimum, which occurs when the effect of LSCATT is the lowest, corresponds to a specific magnitude of *k*. Under our experimental conditions and using the di-4-ANBDQBS dye, *k* was ~2.1 mm on average (n = 24). In detail, for atrial, endocardial, and epicardial stimulations, these values were 2.09 ± 0.09 mm, 2.17 ± 0.10 mm, and 2.0 ± 0.12 mm, respectively (n = 8 for each).

It is also important to note that DWTAP differs from the TAP signal because for DWTAP detection (see Eq 4), all transmurally recorded APs were depth-weighted (see S2D and S2E Fig) using a single exponential decay weighting function with the indicated *k* (Fig 5B) before the APs were averaged. For visualization of the effect of *k* on the DWTAP, we calculated DWTAP for three different values of *k* (Fig 5C). When *k* is high, then DWTAP becomes closer to TAP, while a low value induces a shift in DWTAP away from TAP (to the right).

### Penetration-depth measurements

The data presented above indicate that the *k* in our experiments is a measure of the effects of both the excitation and emission light. In previous studies, a decay constant *k* for the probing depth was calculated from separate measurements of the penetration depths for excitation and emission light. Therefore, to ensure the validity of our new approach for determining *k*, we also performed separate (i.e., control) experiments on cut-open rabbit hearts (see Methods) and measured the penetration depths (see Eqs 1 and 2) for both red excitation (660 nm) and near-infrared emission (735 nm) light. According to our estimates, the penetration depth of the excitation light, *δ*_*ex*_, was 3.15 ± 0.37 mm, and the penetration depth of the emission light, *δ*_*f*_, was 4.46 ± 0.35 mm (n = 5). Using these data, the weighting function was calculated as the product of Eqs 1 and 2 (see Methods). In Fig 5D, the data points of the weighting function are shown. In the same graph, a curve of a single exponential decay function with a constant *k* = 2 mm, which was obtained by fitting the data points of the weighting function, is also presented.

The important fact is that the values for the probing-depth constant determined by both techniques, i.e., our new approach as well as the standard technique, were comparable (2.1 mm versus 2 mm). In addition, the experimental data points of the weighting function were well-approximated with a single exponential decay curve (R^2^ = 0.994). This indicates that the use of a single exponential decay process for probing-depth calculations in our new approach was most likely correct. However, note that the data points at the near epicardial surface do not coincide with the exponential decay curve (see the Discussion).

### Evaluation of OAP upstroke components

**Fig 6:**
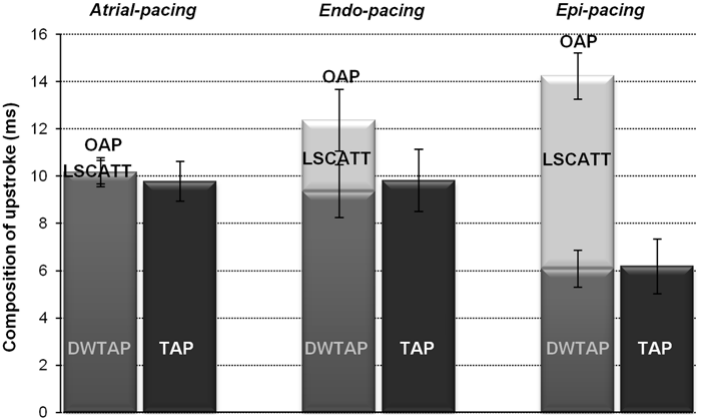
Mean data for the components composing the OAP upstroke. Bars represent the durations (means ± SEMs) for all components that contribute to the OAP upstroke: a transmural AP recorded with a microelectrode versus a full OAP (the sum of the depth-weighted TAP and the lateral-scattering component) obtained with the di-4-ANBDQBS dye, when stimulated in the atrium/endocardium/epicardium. The dark gray bar indicates the TAP, and the gray and light gray bars indicate the DWTAP and LSCATT components, respectively. The durations of the upstroke for TAP, DWTAP, and OAP were calculated using the time points that corresponded to 10 and 90% of the amplitude. LSCATT was calculated as the difference between OAP and DWTAP. Note that LSCATT was significantly different (P <0.05, n = 8 for each) for the various types of pacing.

Fig 6 shows a quantitative evaluation of OAP upstroke components. The mean values for TAP, DWTAP, and LSCATT were 9.79 ± 0.84 ms, 10.18 ± 0.62 ms, and 0.0 ± 0.56 ms, respectively, for atrial pacing, 9.83 ± 1.32 ms, 9.37 ± 1.12 ms, and 3.01 ± 1.30 ms, respectively, for endocardial pacing, and 6.19 ± 1.16 ms, 6.09 ± 0.79 ms, and 8.16 ± 0.98 ms, respectively, for epicardial pacing (n = 8 for each).

Under our experimental conditions, the range of depths from which TAP was obtained and the depth range that produces 90% of the fluorescence probing depth for the di-4-ANBDQBS dye were up to 4.92 ± 0.28 mm and 4.83 ± 0.18 mm (n = 24), respectively, from the epicardium. In addition, assuming that the LSCATT component of the OAP upstroke corresponds to the part of the wave that is parallel to the epicardium, we calculated the width of the scattering volume (by multiplying the observed value of LSCATT and the conduction velocity, which was taken to be 0.5 m/s) that caused the OAP. During epi-pacing, when the LSCATT component was the largest, the width of the scattering volume was 4.1 ± 0.5 mm (n = 8).

Finally, we also calculated τ_opt_, and the calculations yielded values of 5.16 ± 0.60, 4.87 ± 0.33, and 6.4 ± 0.78 for atrial, endocardial, and epicardial stimulations, respectively (n = 8 for each).

## Discussion

To date, cardiac electrical activity has not commonly been monitored using an approach combining OM and transmural glass-microelectrode recordings because of the difficulty in performing such experiments on the whole heart. Our present work differs from earlier studies in that the measurement of the transmural AP upstroke profile, along with the di-4-ANBDQBS dye, allowed us to obtain more objective information about the effect of the excitation-wave propagation on the formation of the OAP upstroke. Our key findings are as follows:

1. Our analyses of the transmural AP enabled us to determine the dependence of the AP activation time on the depth in the LV wall, revealing both the electrical wave-propagation and the degree of electrical inhomogeneity in the heart. This electrical inhomogeneity is apparently related to the morphological inhomogeneity of the myocardium (fiber orientation, intercellular connections, fats, blood vessels, and other factors) [10,23];
2. The simultaneous recordings of a transmural AP and an OAP allowed us to determine the probing-depth constant of the fluorescence measurement, which evaluates the distribution of both the excitation light and the emission light in the myocardium, during each experiment. This procedure differs from that in other studies because we directly estimated the fluorescence light distribution in the myocardium during the OM experiment. In contrast, experiments conducted by other researchers, as well as our experiments described in a different section of the manuscript, estimated the fluorescence light distribution from separate measurements of the penetration of the excitation light and of the emission light in the myocardium [1,4,6,16,24];
3. When using the calculated transmural AP upstroke, we were able to split the OAP upstroke into two components: DWTAP and LSCATT.

These discoveries were possible because of our new approach, in which the actual profile of the transmural electrical AP upstroke across the LV wall is determined simultaneously with the OAP upstroke. This procedure reveals the electrical inhomogeneity of the heart; however, this inhomogeneity was not examined in detail, as we focused mainly on evaluating the composition of the OAP upstroke.

Previous studies [8] have reported that the oncoming lateral component of electrical wave forms the LSCATT component of the OAP, and if there is no lateral propagation, then the LSCATT also does not exist. Using this knowledge and by selecting various experimental conditions that create conditions for excitation-propagation that could potentially appear naturally in the myocardium, we managed to split the OAP upstroke into two components (DWTAP and LSCATT). Our results demonstrate that after the transmural AP (TAP) upstroke is corrected for the probing depth of the fluorescence measurement, the duration (note: not the shape) of the obtained DWTAP upstroke is not changed significantly. This can be due to the fact that both the thickness of the LV of rabbit heart and the probing depth of fluorescence under our experimental conditions were of almost the same magnitude. The depth range at which TAP was obtained from epi-to-subendocardium was up to 4.92 mm, and 90% of the collected fluorescence originated up to a depth of 4.83 mm from the epicardium. This indicates that the di-4-ANBDQBS dye was able to provide a detectable signal throughout almost the entire thickness of the rabbit LV wall (~5.1 mm). However, as shown in Fig 4, the DWTAP upstroke shape highly coincides with the OAP only during atrial pacing because under such conditions, LSCATT is almost negligible. This result confirms the assumption that when LSCATT is absent, the upstrokes of DWTAP and OAP should be equal. It should be noted that the difference between the OAP and DWTAP upstrokes revealed the magnitude of the LSCATT component, which was highly dependent on the stimulation type. In contrast to atrial stimulation, the magnitude of the LSCATT component was markedly increased when stimulation from the endocardium or from the epicardium was used. Thus, under such experimental conditions, alongside the electrical wave propagating from the endocardial-to-epicardial surface, which occurs with atrial pacing, the propagation parallel to the epicardial surface appears as well. Obviously, the greatest magnitude of LSCATT was obtained with epicardial stimulation, possibly due to the wavefront that was almost perpendicular to the epi-surface. Our data indicate that during epi-stimulation, only approximately half of the OAP upstroke is derived from the depth-weighted electrical signal (i.e., DWTAP). Thus, the remaining part of the OAP upstroke can be attributed to LSCATT, whose duration was prolonged by ~8 ms on average. Moreover, if the wavefront were completely perpendicular to the epi-surface, then only one component (LSCATT) would form the entire upstroke of the OAP.

Simulation studies indicate that the OAP upstroke could be longer than the epi-AP by a factor of up to ~9 [5]. In accordance with previous experimental [16] and simulation [1,5,8] studies, our data demonstrate that the prolongation of the OAP upstroke duration arises not only from the averaged, depth-weighted electrical signal but also from the lateral scattering component. In addition, in OM experiments, the medium surrounding the heart influences the amount of photon scatting out from the tissue near the surface, as well as the OAP upstroke duration [1,5]. Under our experimental conditions, when an air-tissue interface was used, the OAP upstroke could be enlarged by approximately 5% compared with glass-tissue boundary conditions [5], i.e., the interface most often used in various studies.

In this study, to evaluate the influence of both DWTAP and LSCATT on the OAP upstroke shape, we needed to determine the probing-depth constant of the fluorescence measurement. According to diffusion theory, photon transport away from a light source is followed by an exponential decay, and the optical signal recorded from the epicardial surface is a result of the distribution of the excitation and emission light in the myocardium [19]. This assumption allowed us to evaluate the probing-depth constant (*k*) of the fluorescence measurement. When calculating that constant, we assumed that the probing of the fluorescence in the tissue could be described by a single exponential decay function. The correctness of this assumption was further verified in separate experiments in which the penetration depths of excitation and emission light were measured; indeed, a monoexponential function fit the experimental data very well (R^2^ = 0.994). A similar approach has been used by other investigators as well and has demonstrated that a single exponential decay function can be use to approximate the distribution of the photon density in the three-dimensional volume of the ventricle after uniform epicardial illumination [5]. Importantly, the values of *k* obtained under routine experiments using our new approach and in separate (control) measurements of comparable fluorescent light (a product of the excitation and emission light) were almost of the same magnitude (2.1 mm versus 2.0 mm, respectively). It should be noted that the values of *k* obtained under our experimental conditions using a long-wavelength dye (di-4-ANBDQBS) were almost twice those obtained using short-wavelength dyes (blue/green) in previous studies [5].

Slight changes in the magnitude of *k* were found even in the same heart for measurements at different places in the LV wall, apparently due to the optical properties of the tissue, although these changes were negligible. In general, the fiber orientation should not change the optical properties of myocardial cells, but structures between the cell layers (fats, connecting tissues, vessels, etc.) could have dissimilar optical properties and thus could influence the value of *k*. In thin tissue, (such as the tissue in the rabbit right ventricle or atrium) and/or in a small heart (rat, guinea-pig), it would apparently not be possible to determine this constant precisely using di-4-ANBDQBS as a dye because a biopsy should be at least 75-100 optical depths (excitation wavelengths) [25]. The rabbit heart (LV thickness ~5 mm) could be at the limit of the usable sample size for 660 nm illumination.

The di-4-ANBDQBS dye was able to provide a detectable fluorescence signal up to a depth of 4.83 mm from the epicardium. This value for the probing depth of a fluorescence measurement is much larger than those estimated for blue/green dyes, whose probing depths have varied from 0.3 to 2 mm in different studies [4,6,16,23,24]. Possible reasons for the differences in the estimates of depth-weighted optical signals may include influences from many factors, e.g., the experimental setup, the dye used, differences in species, unfocussed light, the illumination geometry, etc. [5]. Certainly, all of these factors can affect the optical signal, although it is very difficult to quantify their effects. In general, our OM experimental conditions and the conditions used in other OM studies were comparable, and the calculated the width of the scattering volume (~4 mm) is consistent with a previously reported value (~1-2 mm radially) [6]; the calculated value of τ_opt_ (~5) is also in agreement with the value reported in previous studies [8].

In additional control experiments, we determined the penetration depths of excitation light (660 nm) and of emission light (735 nm). Penetration depth constants detected in a pig heart using analogous wavelengths were reported as 2.56 mm and 3.3 mm for excitation and emission light, respectively [26]. The probing depth constants in our study were approximately 25% larger for both wavelengths than the analogous values in that study, again because of experimental differences.

By combining OM with computer simulation, other studies have investigated the OAP upstroke morphology to predict the subsurface orientation of the wavefront [8,18]. In these experiments, the position of dV/dt_max_ in the optical signal upstroke (in the studies [8] labeled as V_f_*) was used as an indicator of the direction of wave propagation. In principle, this procedure allows an evaluation of the direction of propagation for simplified heart model geometries (without any inhomogeneities) using a monodomain representation [8,18]. However, conducting a realistic simulation using a bidomain model [5] and considering more complex situations, e.g., an inhomogeneous morphology of the heart, together with a surrounding bath, the position of the maximal slope of the upstroke may not always be informative. It is important to note that the information that can be obtained may be limited when short-wavelength light (in the case of the di-4-ANEPPS dye), which does not penetrate deep into the tissue, is used. Under such circumstances, even small subepicardial inhomogeneities could be particularly important and could affect the results. Meanwhile, when using a longer wavelength dye, such as di-4-ANBDQBS, these small inhomogeneities have a smaller influence on the overall optical signal. This is due to the fact that under such conditions, the fluorescence light can be collected not only at the surface but also, as was demonstrated in this study, as deep as up to 4.83 mm from the epicardium. Therefore, optical signals average all local inhomogeneities throughout the entire LV wall thickness; consequently, they can smooth out the impact of subepicardial inhomogeneities on the upstroke. Accordingly, in such circumstances, tissue looks effectively more homogeneous, and the resulting data could be similar to the data obtained using a simplified heart model. Of note, a very recent study demonstrates a substantial influence of coronary vessels on the OAP upstroke shape [27].

However, in addition to tissue inhomogeneities, other factors, such as electrical/optical boundary features, may also influence V_f_*. In our study, the optical boundary effect, which demonstrates the inadequacy of the exponential decay function for describing the photon distribution at the epicardial surface, can be seen in Fig 5D. The well-known electrotonic electrical boundary effect at the epicardial surface [12] can also be observed in our experimental data collected from the microelectrode recordings for atrial pacing when measuring the rise time of the AP upstroke transmurally (a bend at a distance of up to 500 μm from the epicardium can be seen in S3 Fig). However, it is possible that optical and electrical boundary effects, due to their opposite actions, could potentially mask each other in the upstroke of the OAP. Only a detailed analysis of both optical and electrical boundary effects may reveal how these processes could influence the V_f_* with different types of pacing. Once again, these data show that V_f_* may not always be informative. Therefore, it is important to look for other parameters of the upstroke of the OAP that could help to evaluate features of the electrical wave propagation and/or of the morphological structure of the myocardium. According to our data, the ratio of LSCATT to DWTAP can provide an averaged angle of the wavefront orientation and can serve as a simplified indication of the direction in which the electrical wave is propagating. In addition, we can propose that an approximation of the OAP upstroke using a multiplex fitting curve could potentially help to analyze OM data as well.

Together with previous investigations, our study demonstrates that the OAP upstroke is influenced by various factors. It is thus clear that the OAP upstroke contains information about the peculiarities of electrical excitation and wave propagation in the heart, although it remains unclear how to extract this data.

The procedure used in our study for calculating parameters such as the probing-depth constant and both the depth-weighted and lateral-scattering components, could be used with different experimental conditions, e.g., at short or long distances from the pacing electrode or with different excitation wavelengths and/or dyes. Thus, this technique creates new ways to investigate and evaluate excitation-propagation properties in future OM experiments.

## Conclusions

The co-registration of OM and transmural microelectrode APs enabled the probing depth of fluorescence measurements to be calculated and the OAP upstroke to be divided into two components (depth-weighted and lateral-scattering), and it also allowed the relative strengths of their effects on the shape of the OAP upstroke to be evaluated. We believe that these data may shed light on the use of the OAP upstroke shape as a tool for the examination of excitation wave propagation in whole heart preparations, for which data remain limited.

## Limitations

Optical/electrical signals interfere with mechanical contractions; therefore, the heart tissue was immobilized using uncoupling drugs [28]. In addition, we did not record APs from the actual endocardial surface; instead, we recorded APs from the subendocardium because the thickness of the LV wall was always measured only after the termination of the experiment.

## Supporting Information

S1 Fig The OAP signal measured using different pixel areas. (A) The overlapped original records of the OAP obtained from 1 x 1 (gray) and 5 x 5 (black) pixels, acquired at a frame rate of 2000 Hz and 500 Hz, respectively. (B) The same data as in (A) but on an expanded time scale. The raw data (without filtering) are shown for 5 x 5 pixels recordings. The OAP from 1 x 1 pixels was obtained by averaging of 12 signals. (C-D) OAP and its upstroke on an expanded time scale before (gray) and after (black) being fit with a 5-parameter logistic function. (TIF)
(TIF)

S2 Fig Transmural distribution of the AP upstroke. **(**A-B) Normalized APs and their upstrokes on an expanded time scale; the APs were recorded in cells located at different transmural depths. (C) The average of the data from the upstrokes of all action potentials from transmural recordings of the LV wall (between the epicardium and the endocardium). (D-E) The same electrical APs and their upstrokes on an expanded time scale (as in A-B) but weighted with a single exponential decay function (*exp*(*-z*/*k*)) when *k* = 2.1. (F) The averaged depth-weighted transmural AP upstroke (dashed line) versus TAP (dotted line). (TIF)
(TIF)

S3 Fig Rise time of the AP upstroke. The change in the electrical AP upstroke rise time (calculated from 10% to 90% of the AP amplitude) as a function of the LV wall depth during atrial pacing. Note that an electrical boundary effect is visible (as a reduced AP upstroke rise time) in subepicardial cells up to ~0.5 mm. (TIF)
(TIF)

S1 Table The mean values of standard AP parameters. (doc)
(DOC)

## References

[1] Bishop MJ, Rodriguez B, Qu F, Efimov IR, Gavaghan DJ, Trayanova NA. The role of photon scattering in optical signal distortion during arrhythmia and defibrillation. Biophys J 2007;93: 3714–3726. 17978166 10.1529/biophysj.107.110981 PMC2072057

[2] Kong W, Ideker RE, Fast VG. Intramural optical mapping of V(m) and Ca(i)2+ during long-duration ventricular fibrillation in canine hearts. Am J Physiol Heart Circ Physiol 2012;302: H1294–1305. 10.1152/ajpheart.00426.2011 22268104 PMC3311483

[3] Nanthakumar K, Jalife J, Masse S, Downar E, Pop M, Asta J, et al Optical mapping of Langendorff-perfused human hearts: establishing a model for the study of ventricular fibrillation in humans. Am J Physiol Heart Circ Physiol 2007;293: H875–880. 10.1152/ajpheart.01415.2006 17369453

[4] Baxter WT, Mironov SF, Zaitsev AV, Jalife J, Pertsov AM. Visualizing excitation waves inside cardiac muscle using transillumination. Biophys J 2001;80: 516–530. 11159422 10.1016/S0006-3495(01)76034-1 PMC1301253

[5] Bishop MJ, Rodriguez B, Eason J, Whiteley JP, Trayanova N, Gavaghan DJ. Synthesis of voltage-sensitive optical signals: application to panoramic optical mapping. Biophys J 2006;90: 2938–2945. 16443665 10.1529/biophysj.105.076505 PMC1414570

[6] Ding L, Splinter R, Knisley SB. Quantifying spatial localization of optical mapping using Monte Carlo simulations. IEEE Trans Biomed Eng. 2001;48: 1098–1107. 11585033 10.1109/10.951512

[7] Walton RD, Mitrea BG, Pertsov AM, Bernus O. A novel near-infrared voltage-sensitive dye reveals the action potential wavefront orientation at increased depths of cardiac tissue. Conf Proc IEEE Eng Med Biol Soc 2009;2009: 4523–4526. 10.1109/IEMBS.2009.5334106 19964642 PMC2895625

[8] Hyatt CJ, Mironov SF, Wellner M, Berenfeld O, Popp AK, Weitz DA, et al Synthesis of voltage-sensitive fluorescence signals from three-dimensional myocardial activation patterns. Biophys J 2003;85: 2673–2683. 14507730 10.1016/s0006-3495(03)74690-6 PMC1303491

[9] Warren M, Spitzer KW, Steadman BW, Rees TD, Venable P, Taylor T, et al High-precision recording of the action potential in isolated cardiomyocytes using the near-infrared fluorescent dye di-4-ANBDQBS. Am J Physiol Heart Circ Physiol 2010;299: H1271–1281. 10.1152/ajpheart.00248.2010 20601458 PMC2957348

[10] Knisley SB, Neuman MR. Simultaneous electrical and optical mapping in rabbit hearts. Ann Biomed Eng 2003;31: 32–41. 12572654 10.1114/1.1535413

[11] Walton RD, Smith RM, Mitrea BG, White E, Bernus O, Pertsov AM. Extracting surface activation time from the optically recorded action potential in three-dimensional myocardium. Biophys J 2012;102: 30–38. 10.1016/j.bpj.2011.10.036 22225795 PMC3250680

[12] Kelly A, Ghouri IA, Kemi OJ, Bishop MJ, Bernus O, Fenton FH, et al Subepicardial action potential characteristics are a function of depth and activation sequence in isolated rabbit hearts. Circ Arrhythm Electrophysiol 2013;6: 809–817. 10.1161/CIRCEP.113.000334 23733913

[13] Di Diego JM, Sicouri S, Myles RC, Burton FL, Smith GL, Antzelevitch C. Optical and electrical recordings from isolated coronary-perfused ventricular wedge preparations. J Mol Cell Cardiol 2013;54: 53–64. 10.1016/j.yjmcc.2012.10.017 23142540 PMC3535682

[14] Masse S, Downar E, Chauhan V, Sevaptsidis E, Nanthakumar K. Ventricular fibrillation in myopathic human hearts: mechanistic insights from in vivo global endocardial and epicardial mapping. Am J Physiol Heart Circ Physiol. 2007;292: H2589–2597. 17259437 10.1152/ajpheart.01336.2006

[15] Efimov IR, Huang DT, Rendt JM, Salama G. Optical mapping of repolarization and refractoriness from intact hearts. Circulation 1994;90: 1469–1480. 10.1161/01.CIR.90.3.1469 8087954

[16] Girouard SD, Laurita KR, Rosenbaum DS. Unique properties of cardiac action potentials recorded with voltage-sensitive dyes. J Cardiovasc Electrophysiol 1996;7: 1024–1038. 8930734 10.1111/j.1540-8167.1996.tb00478.x

[17] Kanaporis G, Martisiene I, Jurevicius J, Vosyliute R, Navalinskas A, Treinys R, et al Optical mapping at increased illumination intensities. J Biomed Opt 2012;17: 96007–96001. 10.1117/1.JBO.17.9.096007 23085908 PMC3602814

[18] Hyatt CJ, Mironov SF, Vetter FJ, Zemlin CW, Pertsov AM. Optical action potential upstroke morphology reveals near-surface transmural propagation direction. Circ Res 2005;97: 277–284. 10.1161/01.RES.0000176022.74579.47 15994436

[19] Gardner CM, Jacques SL, Welch AJ. Light transport in tissue: Accurate expressions for one-dimensional fluence rate and escape function based upon Monte Carlo simulation. Lasers Surg Med 1996;18: 129–138. 8833281 10.1002/(SICI)1096-9101(1996)18:2<129::AID-LSM2>3.0.CO;2-U

[20] Svaasand LO. Optical dosimetry for direct and interstitial photoradiation therapy of malignant tumors. Prog Clin Biol Res 1984;170: 91–114. 6531373

[21] Gilmour RF Jr, Evans JJ, Zipes DP. Purkinje-muscle coupling and endocardial response to hyperkalemia, hypoxia, and acidosis. Am J Physiol 1984;247: H303–311. 6465334 10.1152/ajpheart.1984.247.2.H303

[22] Spach MS, Heidlage JF, Dolber PC, Barr RC. Extracellular discontinuities in cardiac muscle: evidence for capillary effects on the action potential foot. Circ Res 1998;83: 1144–1164. 9831709 10.1161/01.res.83.11.1144

[23] Knisley SB. Transmembrane voltage changes during unipolar stimulation of rabbit ventricle. Circ Res 1995;77: 1229–1239. 7586236 10.1161/01.res.77.6.1229

[24] Efimov IR, Mazgalev TN. High-resolution, three-dimensional fluorescent imaging reveals multilayer conduction pattern in the atrioventricular node. Circulation 1998;98: 54–57. 9665060 10.1161/01.cir.98.1.54

[25] Welch AJ, Gardner C, Richards-Kortum R, Chan E, Criswell G, Pfefer J, et al Propagation of fluorescent light. Lasers Surg Med 1997;21: 166–178. 9261794 10.1002/(sici)1096-9101(1997)21:2<166::aid-lsm8>3.0.co;2-o

[26] Mitrea BG, Wellner M, Pertsov AM. Monitoring intramyocardial reentry using alternating transillumination. Conf Proc IEEE Eng Med Biol Soc 2009;2009: 4194–4197. 10.1109/EMBS.2009.5334048 19964628 PMC2895618

[27] Bishop MJ, Plank G. Simulating photon scattering effects in structurally detailed ventricular models using a Monte Carlo approach. Front Physiol 2014;5: 338 10.3389/fphys.2014.00338 25309442 PMC4164003

[28] Fedorov VV, Lozinsky IT, Sosunov EA, Anyukhovsky EP, Rosen MR, Balke CW, et al Application of blebbistatin as an excitation-contraction uncoupler for electrophysiologic study of rat and rabbit hearts. Heart Rhythm 2007;4: 619–626. 17467631 10.1016/j.hrthm.2006.12.047

