# Supplementary figures and images for: Evaluation of Excitation Propagation in the Rabbit Heart: Optical Mapping and Transmural Microelectrode Recordings
Source: PLoS One. 2015 Apr 16;10(4):e0123050. doi: 10.1371/journal.pone.0123050 (PMC4400155; doi:10.1371/journal.pone.0123050)

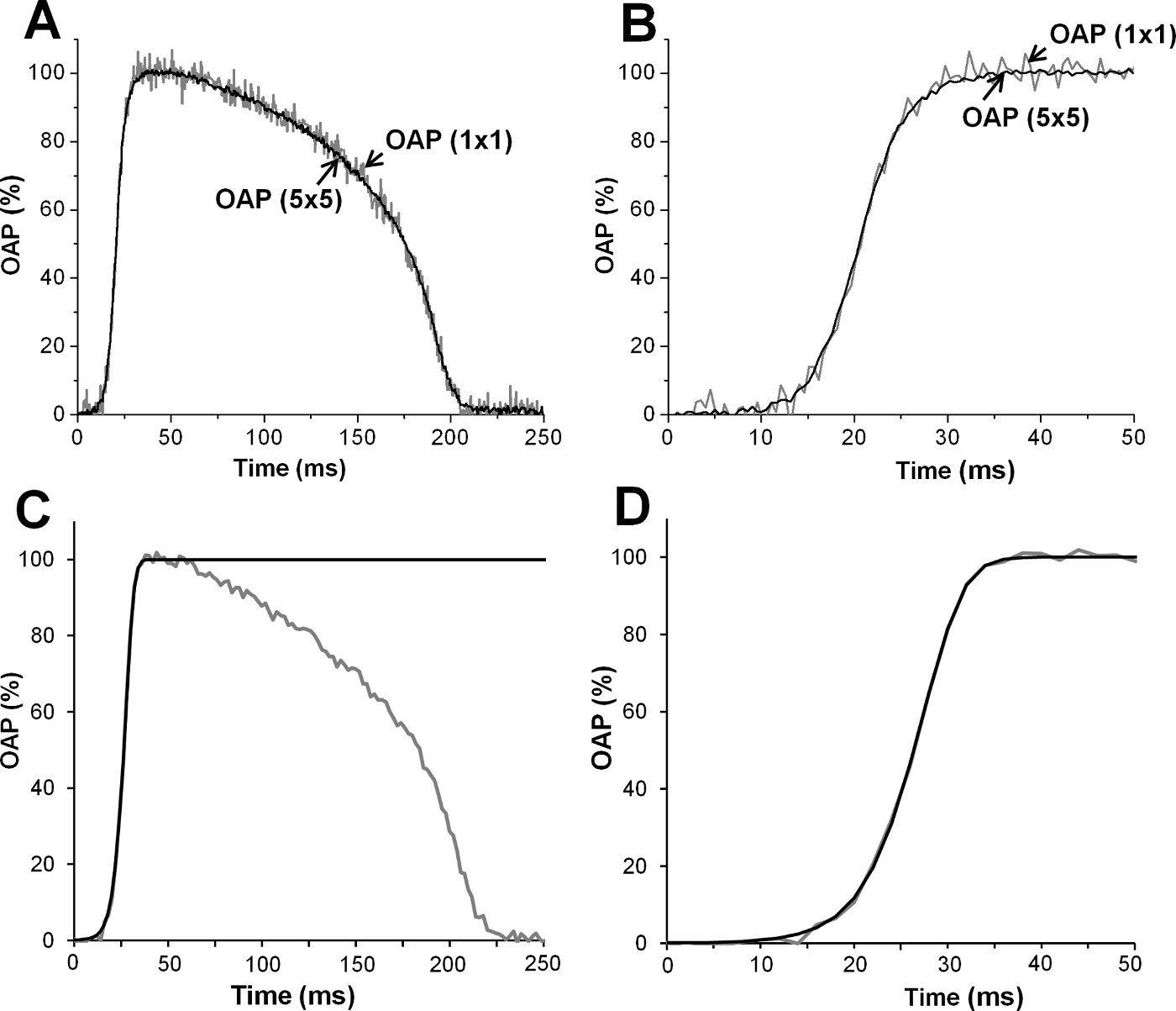

Supplement: S1 Fig — (A) The overlapped original records of the OAP obtained from 1 x 1 (gray) and 5 x 5 (black) pixels, acquired at a frame rate of 2000 Hz and 500 Hz, respectively. (B) The same data as in (A) but on an expanded time scale. The raw data (without filtering) are shown for 5 x 5 pixels recordings. The OAP from 1 x 1 pixels was obtained by averaging of 12 signals. (C-D) OAP and its upstroke on an expanded time scale before (gray) and after (black) being fit with a 5-parameter logistic function. (TIF) (TIF) [file pone.0123050.s001.tif]

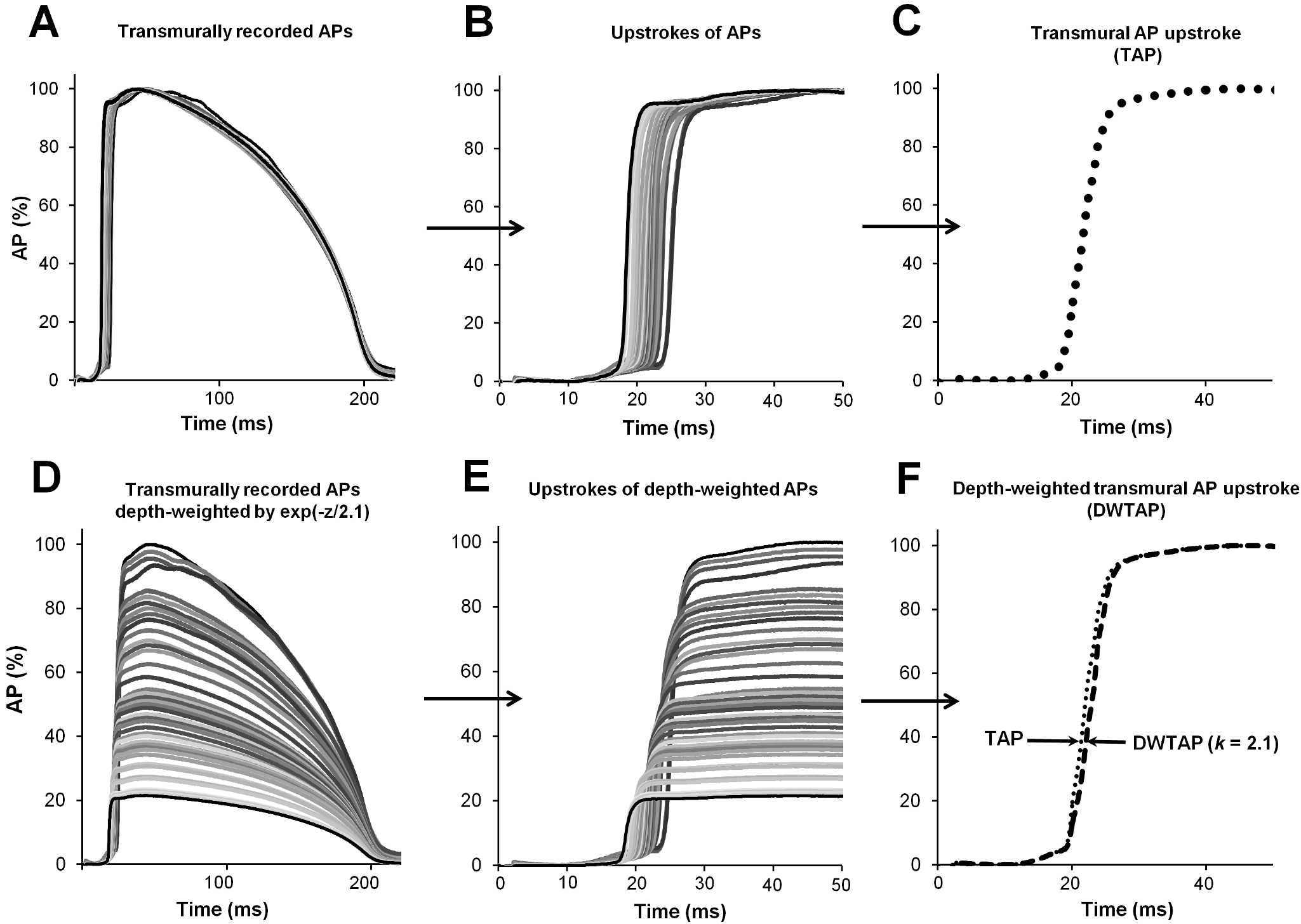

Supplement: S2 Fig — (A-B) Normalized APs and their upstrokes on an expanded time scale; the APs were recorded in cells located at different transmural depths. (C) The average of the data from the upstrokes of all action potentials from transmural recordings of the LV wall (between the epicardium and the endocardium). (D-E) The same electrical APs and their upstrokes on an expanded time scale (as in A-B) but weighted with a single exponential decay function (exp(-z/k)) when k = 2.1. (F) The averaged depth-weighted transmural AP upstroke (dashed line) versus TAP (dotted line). (TIF) (TIF) [file pone.0123050.s002.tif]

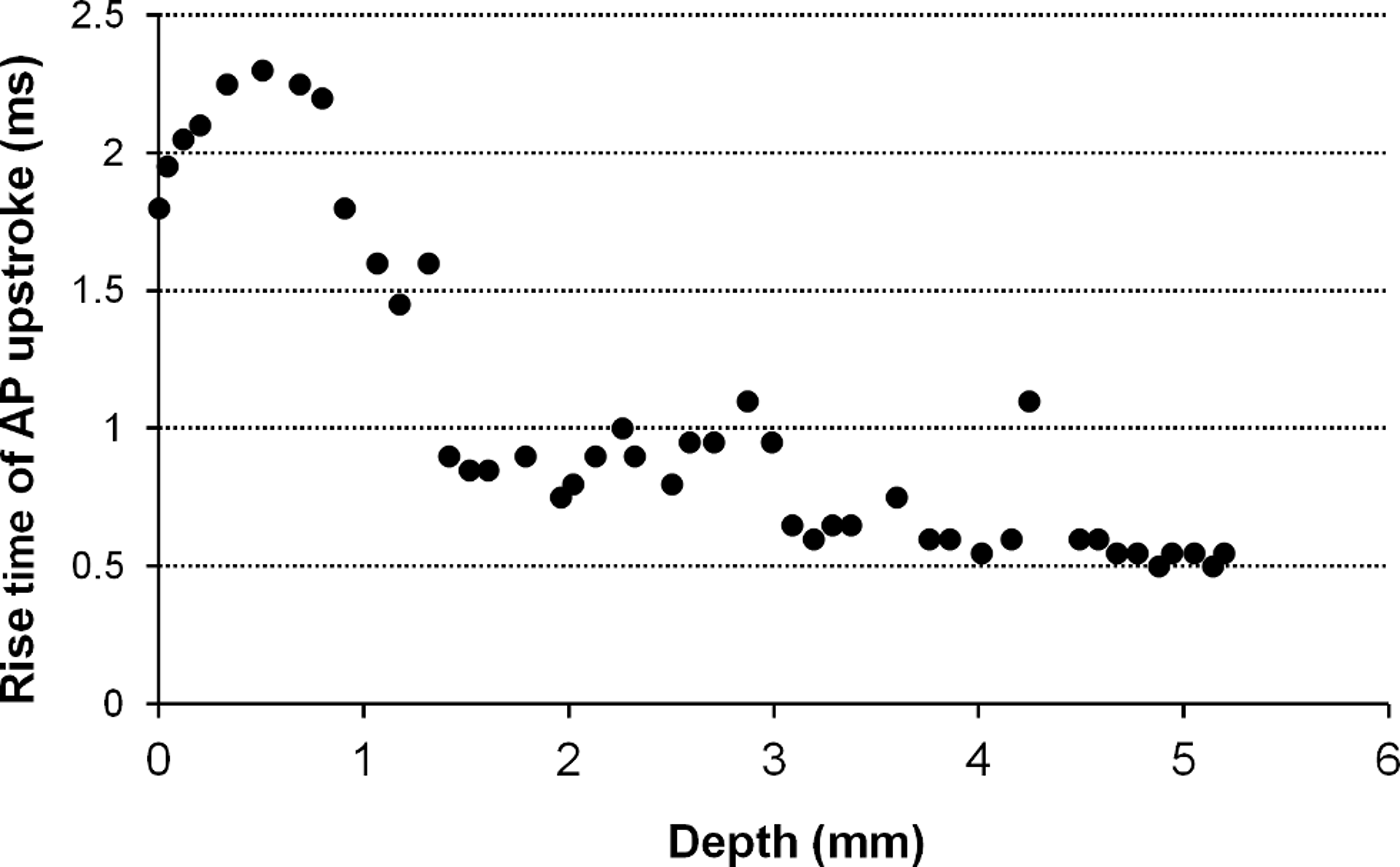

Supplement: S3 Fig — The change in the electrical AP upstroke rise time (calculated from 10% to 90% of the AP amplitude) as a function of the LV wall depth during atrial pacing. Note that an electrical boundary effect is visible (as a reduced AP upstroke rise time) in subepicardial cells up to ~0.5 mm. (TIF) (TIF) [file pone.0123050.s003.tif]
