# Supplementary material for: Evaluation of Excitation Propagation in the Rabbit Heart: Optical Mapping and Transmural Microelectrode Recordings
Source: PLoS One. 2015 Apr 16;10(4):e0123050. doi: 10.1371/journal.pone.0123050 (PMC4400155; doi:10.1371/journal.pone.0123050)
Supplement: S1 Table — (doc) (DOC) [file pone.0123050.s004.doc]

***S1 Table. The mean data of standard AP parameters***

|  | RMP  (mV) | APA  (mV) | dV/dt_max_ (V/s) | APD50  (ms) | APD90  (ms) |
| --- | --- | --- | --- | --- | --- |
| ***Atrium-pacing*** |  |  |  |  |  |
| OAP | - | - | - | 154.4±1.4 | 180.2±0.6 |
| Subendo-AP | ‑82.5±0.6 | 106.9±1.6 | 206.2±23.9* | 151.3±2.0* | 184.2±1.9*† |
| Epi-AP | -80.7±1.3 | 106.1±1.6 | 108.4±19.2 | 143.8±3.6† | 177.0±2.6 |
| ***Endo-pacing*** |  |  |  |  |  |
| OAP | - | - | - | 152.7±1.3 | 181.1±1.1 |
| Subendo-AP | ‑82.7±1.5 | 108.4±2 | 239.7±18.3* | 149.4±3 | 185.3±2.2*† |
| Epi-AP | -79.2±1.2 | 104.5±1.7 | 86.7±16.1 | 142.4±5.2† | 175.5±3.7† |
| ***Epi-pacing*** |  |  |  |  |  |
| OAP | - | - | - | 153.0±2.5 | 182.7±2.1 |
| Subendo-AP | ‑81.8±1.4 | 107.4±1.4 | 250.7±25.7* | 152.5±3.0 | 186.3±2.1*† |
| Epi-AP | -80.0±1.4 | 105.6±1.3 | 102.1±19.7 | 145.0±5.4 | 179.0±3.8 |
|  |  |  |  |  |  |

AP – electrical action potential, APA – action potential amplitude, APD50 and APD90 – action potential duration at 50% and 90% of repolarization, respectively, dV/dt_max_ – maximal value of the first time derivative of the AP upstroke, OAP – optical action potential, RMP – resting membrane potential. *P<0.05 versus Epi-AP, †*P*<0.05 versus OAP for each pacing type, n=9.
